# An Adaptive Unscented Kalman Ilter Integrated Navigation Method Based on the Maximum Versoria Criterion for INS/GNSS Systems

**DOI:** 10.3390/s25113483

**Published:** 2025-05-31

**Authors:** Jiahao Zhang, Kaiqiang Feng, Jie Li, Chunxing Zhang, Xiaokai Wei

**Affiliations:** 1 National Key Laboratory of Photoelectric Dynamic Testing Technology and Instrument in Extreme Environment, North University of China, Taiyuan 030051, China; sz202306192@st.nuc.edu.cn (J.Z.); lijie@nuc.edu.cn (J.L.); s202206050@st.nuc.edu.cn (C.Z.); 2 Avic Taiyuan Aero-Instruments Co., Ltd., Taiyuan 030000, China; 3 School of Electronic Information Engineering, Inner Mongolia University, Hohhot 010021, China; weixiaokai@imu.edu.cn; 4 Inner Mongolia Key Laboratory of Intelligent Communication and Sensing and Signal Processing, Inner Mongolia University, Hohhot 010021, China

**Keywords:** INS/GNSS-integrated navigation, non-Gaussian noise, maximum versoria criterion, unscented Kalman filter

## Abstract

Aimed at the problem of navigation performance degradation in inertial navigation system/global navigation satellite system (INS/GNSS)-integrated navigation systems due to measurement anomalies and non-Gaussian measurement noise in complex navigation environments, an adaptive unscented Kalman filter (AUKF) algorithm based on the maximum versoria criterion (MVC) is developed. The proposed method is designed to enhance INS/GNSS-integrated navigation system robustness and accuracy by addressing the limitations of conventional filtering approaches. An adaptive unscented Kalman filter is constructed to enable dynamic adjustment of filter parameters, allowing for real-time adaptation to measurement anomalies. This ensures accurate tracking of navigation parameter states, thereby improving the robustness of the INS/GNSS-integrated navigation system in the presence of abnormal measurements. On this basis, fully considering the high-order moments of estimation errors, the maximum versoria criterion is introduced as the optimization criterion to construct a novel cost function, further effectively suppressing deviations caused by non-Gaussian disturbances and improving system navigation accuracy. The effectiveness of the proposed method was verified through vehicle navigation experiments. The experimental results demonstrate that the proposed method outperforms traditional approaches, effectively handling measurement anomalies and non-Gaussian measurement noise while maintaining robust navigation performance. Specifically, compared to the EKF, UKF, and MCCUKF, the proposed method reduces the root mean square error of velocity and position by over 60%, 50%, and 30%, respectively, under complex navigation conditions. The algorithm exhibits good accuracy and stability in complex environments, showcasing its practical applicability in real-world navigation systems.

## 1. Introduction

With the rapid development of modern science and technology, navigation technology has gradually become an indispensable core technology in military and civilian fields. In the military field, modern weapons and equipment, such as precision-guided weapons, unmanned aerial vehicle combat systems, and warship formation, have imposed unprecedented requirements for navigational accuracy and reliability [1,2,3]. In the civil field, emerging application scenarios such as intelligent transportation systems and autonomous driving have strict standards for navigational performance. The escalating performance requirements in navigational applications have spurred relentless advancements in navigation technology, simultaneously imposing increasingly stringent demands on the precision, reliability, and robustness of navigation systems. In military applications, the accuracy of navigational systems directly affects combat effectiveness and battlefield survivability. Therefore, developing high-performance and highly reliable navigation systems and technologies has become a hotspot and difficult topic in current research. Traditional single-navigation sensors or systems are no longer able to meet the increasingly complex application requirements [3,4,5,6]. Taking inertial navigation systems (INS) as an example, although they have the advantages of complete autonomy and are not affected by external interference, their errors accumulate over time, making it difficult to meet the requirements of long-term high-precision navigation. The working principle of an INS is based on Newton’s laws of motion, and it calculates positional and attitudinal information by measuring the acceleration and angular velocity of the carrier. However, due to inertial sensor errors (such as zero bias and scale factor errors) and environmental disturbances (such as temperature changes and vibrations), the positioning error of an INS will accumulate over time. On the other hand, although global navigation satellite systems (GNSS) can offer high-precision positioning information, they are susceptible to multipath effects, electromagnetic interference, and other factors [5,6,7], particularly in challenging environments such as urban canyons and underground tunnels, where signal degradation or complete loss frequently occurs. Therefore, combining an INS with a GNSS to form a complementary INS/GNSS-integrated navigation system has become an effective solution to this problem, leveraging the complementary characteristics of both technologies to enhance navigational reliability and accuracy in complex scenarios.

As one of the most common and widely used integrated navigation systems, an IINS/GNSS-integrated navigation system combines the advantages of an INS’s strong autonomy and a GNSS’s high measurement accuracy, and it can provide reliable and effective navigation solutions for different carriers. An INS/GNSS-integrated navigation system is undoubtedly an important component of navigation equipment. However, in practical applications, INS/GNSS-integrated navigation technology still faces numerous challenges. INS/GNSS systems often encounter non-Gaussian system noise from multiple sources. For MEMS inertial sensors, manufacturing imperfections and environmental factors lead to time-varying biases and nonstationary noise characteristics. GNSS measurements in urban environments frequently exhibit heavy-tailed distributions due to inherent defects, such as multipath effects, non-line-of-sight effects, and poor anti-jamming capabilities [4,5,6,7,8,9]. These practical noise characteristics violate the Gaussian assumptions underlying conventional filtering approaches. Signal interference in complex environments is an important factor affecting navigation performance [7,8,9,10]. In addition, the carrier is inevitably affected by external environmental interference during operation (such as high-rise buildings, mountainous terrain, trees, tunnel passages, and electromagnetic interference), resulting in GNSS signal attenuation and even abnormalities [11]. Under such circumstances, GNSS positioning errors may reach tens of meters or even longer, and this measurement information with large errors further exacerbates integrated navigational errors. Furthermore, complex environments are prone to inducing system process uncertainty and non-Gaussian measurement noise disturbances, leading to a decline in the performance of integrated navigation systems [12,13,14,15]. When there is a sudden change in the motion state of the carrier, the system model may not be able to accurately describe the actual motion state, resulting in significant estimation errors in the integrated navigation algorithm; the measurement noise in actual environments may exhibit non-Gaussian characteristics, and traditional filtering algorithms based on Gaussian assumptions have difficulty in accurately describing this noise characteristic. When dealing with non-Gaussian measurement noise, significant estimation errors may occur, leading to a decrease in navigation accuracy.

It is becoming increasingly necessary to obtain accurate navigation information in complex environments [16]. How to effectively integrate INS information and GNSS information to obtain ideal navigation solutions is the key to the effective application of INS/GNSS-integrated navigation systems in complex environments. At present, information fusion algorithms based on Kalman filtering (KF) are widely used in INS/GNSS-integrated navigation technology. However, the application of standard Kalman Filter (KF) or Extended Kalman Filter (EKF) algorithms may lead to filter divergence due to modeling inaccuracies, particularly when dealing with highly nonlinear systems [17,18,19]. This limitation becomes more pronounced in INS/GNSS-integrated navigation systems that incorporate low-cost inertial measurement units (IMUs) with relatively poor performance characteristics. The inherent nonlinearities and noise characteristics of such systems often exceed the estimation capabilities of conventional KF-based approaches, resulting in a suboptimal navigation performance and potential instability in state estimation. The EKF approximates the nonlinear system through the first-order Taylor expansion, which may cause large linearization error and even cause filtering divergence when dealing with strongly nonlinear systems. An INS/GNSS-integrated navigation system usually involves nonlinear system models (such as attitude update equation and position update equation), and an EKF may produce a large linearization error when dealing with these nonlinear models [20]. Although unscented Kalman filters (UKFs) and cubature Kalman filters (CKFs) better handle nonlinear problems through sigma point sampling, they still assume that the measurement noise follows a Gaussian distribution and cannot effectively deal with non-Gaussian measurement noise in practical environments, creating difficulties for these conventional nonlinear filtering approaches in achieving a satisfactory state estimation accuracy, thereby restricting their effectiveness in handling complex noise distributions commonly encountered in practical navigation scenarios [18,19,20]. With the particle filter (PF), while capable of approximating the posterior probability distribution of system states through a large ensemble of particles and demonstrating superior accuracy in handling non-Gaussian measurement noise, its computational complexity increases sharply with the increase in particle numbers, especially in high-dimensional state spaces, where the computational complexity of particle filtering may reach an unacceptable level and its real-time performance is poor [19,20,21,22,23,24,25,26]. The system process uncertainty and measurement noise caused by complex navigation environments, including non-Gaussian and even measurement anomalies, pose certain difficulties for the information fusion and filtering estimation of an INS/GNSS.

Recent advancements in robust filtering for integrated navigation systems have introduced diverse methodologies to address measurement anomalies and non-Gaussian noise. For instance, Hu et al. [27] developed a robust UKF with real-time measurement error detection, which enhances reliability in tightly coupled INS/GNSS systems under hypersonic conditions by adaptively adjusting covariance matrices. Gao et al. proposed a hypothesis-test-constrained Kalman filter and a double-channel sequential probability ratio test, systematically isolating abnormal measurements through statistical thresholds [28,29]. Mahalanobis distance-based methods, such as the robust CKF by Gao et al. automate outlier identification in vehicular navigation by dynamically adjusting noise covariance [30], while Dong et al. [31] extended this approach to underwater scenarios using windowing-based factor graph optimization. Hybrid frameworks, including the set-membership hybrid Kalman filter and quaternion-based adaptive particle filters [32,33], address nonlinear state estimation under systematic uncertainties. Indirect fuzzy CKFs with normalized parameters and model predictive UKFs further improve noise suppression through fuzzy logic and predictive optimization, respectively [34]. For multi-sensor systems, decentralized fusion techniques (e.g., INS/GNSS/CNS integration) and closed-loop covariance feedback CKFs enhance robustness via adaptive mechanisms. Additionally, fading CKFs with Mahalanobis distance criteria and multiple interacting model UKFs demonstrate improved adaptability to time-varying noise in hypersonic environments [35,36,37,38,39,40,41]. While these methods achieve significant progress, many rely on manual threshold tuning, assume prior noise statistics, or incur high computational costs.

In recent years, a Kalman filter algorithm called MCC-KF was proposed, which adopts the maximum correlation entropy criterion (MCC) method for the traditional Kalman filter framework. Hou et al. [42] applied the MCC to a CNS/SINS-integrated navigation system with a linear state equation and a nonlinear measurement equation to improve the navigational accuracy of ballistic missiles. Liu et al. [43] applied the MCC to the relative state measurement of spacecraft with linear state equations and nonlinear measurement equations to cope with complex noise. Chen et al. [44] took the Gaussian kernel function as the cost function, equated the filtering problem with a linear regression problem, and derived a Kalman filter based on the MCC to deal with non-Gaussian process noise. However, the MCC-based filtering approach is limited by numerical instability and the relatively high computational cost due to the exponential operation of Gaussian kernel functions in the MCC. The maximum versoria criterion (MVC) has recently gained attention as a robust optimization criterion for filtering algorithms, and the versoria function is widely used in adaptive filtering because of its thick-tailed characteristic and the advantage of no exponential operation. For instance, Zhao et al. [45] proposed the MVC-EKF algorithm by combining the MVC with an EKF to handle non-Gaussian condition SINS/GNSS-integrated navigation systems in actual missile environments, demonstrating its superiority over traditional methods in terms of steady-state error reduction and robustness during the nonstationary measurement process. Park et al. [46] presented a robust two-step weighted least squares (WLS) algorithm using the Kalman filter with the MVC for robust localization, and the positioning results demonstrate that the localization performances of the proposed algorithms outperform that of the conventional methods. Related research and experimental verification also indicate that optimizing the Kalman filtering method using the MVC can effectively improve the robustness of the algorithm in complex noise environments.

In response to the aforementioned challenges, this paper proposes an adaptive unscented Kalman filter algorithm based on the MVC, termed the MVCAUKF, aimed at improving the navigational performance of INS/GNSS-integrated navigation systems in complex environments. By constructing a cost function based on the maximum versoria criterion and designing an adaptive unscented Kalman filter to address system process uncertainty and non-Gaussian measurement noise, the navigation performance of INS/GNSS-integrated navigation systems in complex environments is improved. The main contributions of this work are as follows: (1) by introducing the maximum versoria criterion, a new cost function based on the MVC is constructed to better adapt to non-Gaussian measurement noise environments, thereby enhancing the accuracy of state estimation. (2) On the basis of the UKF framework, an adaptive mechanism is introduced to dynamically adjust the filtering parameters in real time to cope with system process uncertainty and improve the robustness of the filtering algorithm.

The remaining part of this paper is arranged as follows: Section 2 introduces the principles of the maximum versoria criterion. Section 3 builds the system model. Section 4 discusses the design of the improved MVCAUKF method in this paper and then Section 5 presents the experimental testing and results. Finally, the conclusions are presented in Section 6.

## 2. Maximum Versoria Criterion

For two random variables, A, B∈ℝ, with a joint probability distribution function, $F_{AB}(a,b)$, their generalized similarity can be defined as follows [13]:

(1) $$\Psi (A,B)=E\left[\kappa (A,B)\right]=\int \kappa (a,b)dF_{AB}(a,b),$$

where $E\left[•\right]$ represents the expectation, and κ(•,•) denotes the shift-invariant Mercer-type positive definite kernel function. In this paper, we adopt the versoria function as the kernel function for the relevant calculations, expressed as follows:

(2) $$\kappa (a,b)=V_{r}(a-b)=\frac{8r^{3}}{4r^{2}+{\left\|a-b\right\|}^{2}}=\frac{2r}{1+{\left((a-b)/2r\right)}^{2}},$$

where the symbol $\left\|•\right\|$ means the Euclidean distance between two random variables, which is the straight-line distance between two points in the input space, and *r* > 0 represents the radius of the circle generated by the versoria function, with the center being (0, *r*). Moreover, as the radius increases, the curve of the versoria function becomes progressively steeper.

From Equation (2), it can be observed that the similarity between the two random variables ***A*** and ***B*** reaches its maximum only when ***A*** = ***B***. By performing a Taylor expansion on Equation (2) and substituting it into Equation (1), the following expression is obtained [14]:

(3) $$\Psi (A,B)=\sum_{n=0}^{\infty }\frac{{\left(-1\right)}^{n}}{{\left(2r\right)}^{2n-1}}E\left[{\left(A-B\right)}^{2n}\right],$$

According to Equation (3), the expected information mentioned above includes all even-order moments of the error between the random variables ***A*** and ***B***, which means that the versoria expectation of the random variable error ***A***−***B*** can obtain higher-order effective information from the measurement data. Compared with the classical minimum mean-square error criterion that only uses the second-order moment information of the measurement, the versoria expectation is more suitable for random systems contaminated by non-Gaussian measurement noise. In addition, in terms of kernel function selection, compared with the commonly used Gaussian kernel function, $\kappa (a,b)=G_{\sigma }(a-b)=\exp\left(-\frac{∥a-b{∥}^{2}}{2\sigma^{2}}\right)$, the versoria function exhibits different characteristics from the Gaussian kernel function. From the perspective of function morphology, the versoria function exhibits better nonlinear response characteristics in the dynamic variation process of the random variable error ***A*** − ***B***. Specifically, when the error ***A*** − ***B*** value is within a large range, the curve of the versoria function changes relatively smoothly, and its decay rate is slower than that of the Gaussian kernel function; when the error ***A*** − ***B*** value is small and approaches 0, the steepness of the versoria function gradually converges to that of the Gaussian kernel function, exhibiting similar local sensitivity characteristics. This characteristic gives the versoria function an advantage in filtering algorithms. From the perspective of algorithm performance, under the premise of ensuring the same convergence rate, the cost function constructed using the versoria function can enable the adaptive filtering algorithm to achieve superior performance indicators [13,14]. This advantage is primarily reflected in the reduction in the steady-state error, which can more effectively balance convergence speed and steady-state accuracy, thereby significantly improving the overall performance of the filtering system. Additionally, the versoria function does not involve exponential operations, and the elimination of such operations reduces the computational load, effectively lowering the computational complexity. This is particularly important for real-time systems and resource-constrained computing environments.

## 3. System Model Construction

The state space model of the INS/GNSS-integrated navigation system can be represented by the following state equation and measurement equation:

(4) $$\left\{\begin{array}{c} x_{k}=f\left(x_{k-1}\right)+w_{k}\\ z_{k}=H_{k}x_{k}+u_{k} \end{array}\right.,$$

where the state equation is nonlinear, while the measurement equation is linear; $x_{k}\in {\mathbb{R}}^{n}$ and $z_{k}\in {\mathbb{R}}^{m}$ represent the state vector and measurement vector of the INS/GNSS-integrated navigation system, respectively; $f\left(•\right)$ is the nonlinear function; $H_{k}\in {\mathbb{R}}^{m\times n}$ is the measurement matrix; $w_{k}$ and $u_{k}$ are the state and measurement noise vectors, respectively; and *k* represents the discrete-time index. Assuming the initial state $x_{0}$, $w_{k}$ and $u_{k}$ of the INS/GNSS-integrated navigation system are independent of each other, and $w_{k}$ and $u_{k}$ satisfy the following statistical properties: $E\left(w_{k}\right)=E\left(u_{k}\right)=0$, $E\left(w_{k}w_{j}^{T}\right)=Q_{k}$, and $E\left(u_{k}u_{j}^{T}\right)=R_{k}$.

In practical navigation systems, the measurement errors of the MEMS inertial devices (e.g., gyroscope drift and accelerometer bias) are not only time-varying but also exhibit non-Gaussian properties due to sensor nonlinearities and environmental disturbances. And the system noise, $w_{k}$, and measurement noise, $u_{k}$, often demonstrate non-Gaussian properties. This necessitates the use of robust filtering criteria beyond traditional Gaussian assumptions.

Taking into account the measurement errors of low-cost MEMS inertial devices, which cannot be ignored, the navigation state and measurement vector of the INS/GNSS-integrated navigation system are established as follows:

(5) $$x=\left[\begin{array}{lllll} \delta \varphi & \delta v & \delta p & \varepsilon & \nabla \end{array}\right],$$

(6) $$z=\left[\begin{array}{c} p_{\mathrm{GNSS}}-p_{\mathrm{INS}}\\ v_{\mathrm{GNSS}}-v_{\mathrm{INS}} \end{array}\right],$$

where δφ, δv, and δp represent the three-dimensional attitude error vector, velocity error vector, and position error vector of the INS, respectively; ε and ∇ denote the gyroscope drift and accelerometer bias in the INS, respectively; $p_{\mathrm{GNSS}}$ and $p_{\mathrm{INS}}$ represent the three-dimensional position information output by the GNSS and INS, respectively; and $v_{\mathrm{GNSS}}$ and $v_{\mathrm{INS}}$ denote the three-dimensional velocity information output by the GNSS and INS, respectively.

## 4. Derivation of the MVCAUKF Algorithm

This paper combines the maximum versoria criterion (MVC) with the adaptive unscented Kalman filter (AUKF) model to derive a new method for solving the adaptive filtering of nonlinear non-Gaussian systems. Similar to the structure of standard Kalman filters, the MVCAUKF is also divided into the following two parts: state prediction and measurement update. The specific steps are as follows:

### 4.1. State Prediction

Calculating the sigma sample points and their weights according to the sampling strategy, the initial state of the system is set to $x_{0}$ and the initial value of the filter is defined as follows:

(7) $$\left\{\begin{array}{l} {\hat{x}}_{0}=E\left[x_{0}\right]\\ P_{0}=E\left[(x_{0}-{\hat{x}}_{0})\cdot {\left(x_{0}-{\hat{x}}_{0}\right)}^{T}\right] \end{array},\right.$$

where ${\hat{x}}_{0}$ is the estimated value of the initial system state value, and $P_{0}$ indicates the initial value of the error covariance matrix.

Selecting 2n + 1 sigma sampling points, the following is obtained:

(8) $$\left\{\begin{array}{l} {\hat{\chi }}_{k}^{\left(0\right)}={\hat{x}}_{k}\\ {\hat{\chi }}_{k}^{\left(i\right)}={\hat{x}}_{k}+\left(\sqrt{\left(n+\lambda \right)P_{k\left(i\right)}}\right),i=1,2,\ldots ,n\\ {\hat{\chi }}_{k}^{\left(i\right)}={\hat{x}}_{k}-\left(\sqrt{\left(n+\lambda \right)P_{k\left(i-n\right)}}\right),i=n+1,n+2,\ldots ,2n \end{array}\right.,$$

where *n* represents the dimension of the system state vector, $\lambda =\alpha^{2}(n+\kappa )-n$ is a scaling parameter for adjusting the approximation accuracy, α denotes the main scaling factor for determining the distribution breadth of sigma points near the prior mean, which generally has a value range of $10^{-4}\leq \alpha \leq 1$, and κ is an optional scaling factor.

In determining the corresponding weight coefficients, the following is used:

(9) $$\left\{\begin{array}{l} W_{c}^{\left(0\right)}=\frac{\lambda }{n+\lambda },W_{c}^{\left(i\right)}=\frac{\lambda }{2(n+\lambda )},i=1,2,\ldots ,n\\ W_{m}^{\left(0\right)}=\frac{1}{n+\lambda }+1-\alpha^{2}+\beta ,W_{m}^{\left(i\right)}=\frac{1}{2(n+\lambda )},i=n+1,n+2,\ldots ,2n \end{array}\right.,$$

where β represents the non-negative weighting coefficients, $W_{c}$ is the variance weighting coefficient for the sigma points, and $W_{m}$ is the mean weighting coefficient for the sigma points.

In the existing traditional UKF algorithm, the weight coefficients are typically set as fixed values, but in practical applications, due to external environmental interference and sensor-related factors, both measurement noise and state noise are often random. To address this issue, based on the UKF, this paper designs an adaptive weight coefficient to adjust the state noise and measurement noise according to the actual situation, thereby suppressing the influence of measurement anomalies and external interference.

The 2n + 1 sigma points are propagated through the state equation to compute the one-step predicted values of the system state vector and the error covariance matrix as follows:

(10) $${\tilde{\chi }}_{k+1|k}^{\left(i\right)}=f\left({\hat{\chi }}_{k}^{\left(i\right)}\right),$$

(11) $${\hat{x}}_{k+1|k}=\sum_{i=0}^{2n}W_{m}^{\left(i\right)}{\tilde{\chi }}_{k+1|k}^{\left(i\right)},$$

(12) $$P_{k+1|k}=\sum_{i=0}^{2n}W_{c}^{\left(i\right)}({\tilde{\chi }}_{k+1|k}^{\left(i\right)}-{\hat{x}}_{k+1|k}){\left({\tilde{\chi }}_{k+1|k}^{\left(i\right)}-{\hat{x}}_{k+1|k}\right)}^{T}+Q_{k},$$

where ${\tilde{\chi }}_{k+1|k}^{\left(i\right)}$ represents the value of the sigma sampling point transformed by the nonlinear state equation, and $Q_{k}$ is the covariance matrix of the system noise at time *k*.

### 4.2. Measurement Update

Based on the one-step predicted values from Equations (11) and (12), new sampling points are generated through the unscented transform as follows:

(13) $$\left\{\begin{array}{l} \chi_{k+1|k}^{\left(0\right)}={\hat{x}}_{k+1|k}\\ \chi_{k+1|k}^{\left(i\right)}={\hat{x}}_{k+1|k}+\left(\sqrt{\left(n+\lambda \right)P_{k\left(i\right)}}\right),i=1,2,\ldots ,n\\ \chi_{k+1|k}^{\left(i\right)}={\hat{x}}_{k+1|k}-\left(\sqrt{\left(n+\lambda \right)P_{k\left(i-n\right)}}\right),i=n+1,n+2,\ldots ,2n \end{array}\right.,$$

Combining Equations (4) and (13), the mean and covariance matrix of the predicted observations are obtained through weighted summation as follows:

(14) $$z_{k+1|k}^{\left(i\right)}=h\left(\chi_{k+1|k}^{\left(i\right)}\right),i=0,1,2,\ldots ,2n,$$

(15) $${\hat{z}}_{k+1|k}=\sum_{i=0}^{2n}W_{m}^{\left(i\right)}z_{k+1|k}^{\left(i\right)},$$

(16) $$P_{(xz),k+1|k}=\sum_{i=0}^{2n}W_{c}^{\left(i\right)}(\chi_{k+1|k}^{\left(i\right)}-{\hat{x}}_{k+1|k}){\left(z_{k+1|k}^{\left(i\right)}-{\hat{z}}_{k+1|k}\right)}^{T},$$

(17) $$P_{(zz),k+1|k}=\sum_{i=0}^{2n}W_{c}^{\left(i\right)}(z_{k+1|k}^{\left(i\right)}-{\hat{z}}_{k+1|k}){\left(z_{k+1|k}^{\left(i\right)}-{\hat{z}}_{k+1|k}\right)}^{T}+R_{k},$$

where $P_{\left(xz\right)}$ is the cross covariance matrix between the state variables and the measurement variables, $P_{\left(zz\right)}$ is the predicted measurement covariance matrix, and $R_{k}$ is the observed noise covariance matrix at time *k*.

Based on Equations (16) and (17), the Kalman gain is updated as follows:

(18) $$K_{k+1}=P_{(xz),k+1|k}P_{(zz),k+1|k}^{-1},$$

Based on Equations (11), (12), (15), and (18), the system state vector and covariance matrix are updated as follows:

(19) $${\hat{x}}_{k+1}={\hat{x}}_{k+1|k}+K_{k+1}\left(z_{k+1}-{\hat{z}}_{k+1|k}\right),$$

(20) $$P_{k+1}=P_{k+1|k}+K_{k+1}P_{(zz),k+1|k}K_{k+1}^{T},$$

The relevant adaptive weight calculation is as follows:

State prediction error:

(21) $$\Delta x_{j}=x_{k+1}-{\hat{x}}_{k+1|k},$$

Measurement error:

(22) $$\Delta z_{j}=z_{k+1}-{\hat{z}}_{k+1|k},$$

(23) $$\left\|\Delta x_{j}\right\|=\sqrt{\Delta x_{j}^{T}\Delta x_{j}},$$

(24) $$\left\|\Delta z_{j}\right\|=\sqrt{\Delta z_{j}^{T}\Delta z_{j}},$$

(25) $$\lambda_{j}=\left\|\Delta x_{j}\right\|\left\|\Delta z_{j}\right\|,$$

(26) $$W_{c}^{\left(i\right)}=W_{m}^{\left(i\right)}=\frac{\lambda_{j}}{\sum_{k=0}^{2n}\left\|\Delta x_{j}\right\|\left\|\Delta z_{j}\right\|},j=1,2,\ldots ,2n,$$

For the system measurement model represented by Equation (4), the error vector is constructed using the state prediction error and measurement error according to Equations (21) and (22), as follows:

(27) $$\xi_{k+1}={\left[\Delta x_{k+1}^{T},\;\Delta z_{k+1}^{T}\right]}^{T},$$

And by performing Cholesky decomposition on the error vector, the square root factor $C_{k+1}$ can be obtained as follows:

(28) $$E\left[\xi_{k+1}\xi_{k+1}^{T}\right]=\left[\begin{array}{cc} P_{k+1} & 0\\ 0 & R_{k+1} \end{array}\right]=\left[\begin{array}{cc} S_{k+1}S_{k+1}^{T} & 0\\ 0 & S_{r,k+1}S_{r,k+1}^{T} \end{array}\right]=C_{k+1}C_{k+1}^{T},$$

Multiplying both sides of the measurement equation in Equation (4) by $C_{k+1}^{-1}$ yields the following form:

(29) $$d_{k+1}=G_{k+1}x_{k+1}+e_{k+1},$$

where

(30) $$\begin{array}{l} d_{k+1}=B_{k+1}^{-1}\left[\begin{array}{c} {\hat{x}}_{k+1|k}\\ z_{k+1}-{\hat{z}}_{k+1|k}+H_{k+1}{\hat{x}}_{k+1|k} \end{array}\right]=\left[\begin{array}{cc} S_{k+1}^{-1} & 0\\ 0 & S_{r,k+1}^{-1} \end{array}\right]\left[\begin{array}{c} {\hat{x}}_{k+1|k}\\ z_{k+1}-{\hat{z}}_{k+1|k}+H_{k+1}{\hat{x}}_{k+1|k} \end{array}\right]\\ =\left[\begin{array}{c} S_{k+1|k}^{-1}{\hat{x}}_{k+1|k}\\ S_{r,k+1}^{-1}\left(z_{k+1}-{\hat{z}}_{k+1|k}+H_{k+1}{\hat{x}}_{k+1|k}\right) \end{array}\right]\;\;\;\;\;\;\;\;\;\;\;\;\;\;\;\;\;\;\;, \end{array}$$

(31) $$G_{k+1}=C_{k+1}^{-1}\left[\begin{array}{c} I_{n_{x}}\\ H_{k+1} \end{array}\right]=\left[\begin{array}{cc} S_{k+1}^{-1} & 0\\ 0 & S_{r,k+1}^{-1} \end{array}\right]\left[\begin{array}{c} I_{n_{x}}\\ H_{k+1} \end{array}\right]=\left[\begin{array}{c} S_{k+1}^{-1}\\ S_{r,k+1}^{-1}H_{k+1} \end{array}\right],$$

(32) $$e_{k+1}=C_{k+1}^{-1}\xi_{k+1},$$

Based on the MVC, the corresponding cost function can be constructed as follows:

(33) $$J_{M}\left(x_{k+1}\right)=\frac{1}{M}\sum_{i=1}^{M}V_{r}\left(e_{k+1}^{\left(i\right)}\right),$$

where $e_{k+1}^{\left(i\right)}$ represents the *i*-th element of $e_{k+1}$, $d_{k+1}^{\left(i\right)}$ denotes the *i*-th element of $d_{k+1}$, and *M* denotes the dimension of $e_{k+1}$ in the manuscript.

Therefore, based on the cost function constructed using the MVC, the problem of the optimal estimation of the navigation state can be transformed into the following cost function minimization problem:

(34) $${\hat{x}}_{k+1|k+1}=\underset{x_{k+1}}{\arg\min}\sum_{i=1}^{M}V_{r}\left(e_{k+1}^{\left(i\right)}\right),$$

The maximum versoria criterion is designed to handle such non-Gaussian measurement noise, where its robust error suppression characteristics provide better performance than traditional quadratic criteria when dealing with outliers and heavy-tailed distributions. The optimal solution for navigation state x can be obtained by taking the derivative of the cost function on $x_{k+1}$, as follows:

(35) $$\frac{𝜕J_{M}\left(x_{k+1}\right)}{𝜕x_{k+1}}=0,$$

After organizing the formula, the following expression is obtained:

(36) $$x_{k+1}={\left(G_{k+1}^{T}D_{k+1}G_{k+1}\right)}^{-1}G_{k+1}^{T}D_{k+1}d_{k+1},$$

where

(37) $$D_{k+1}=\left[\begin{array}{cc} D_{x,k+1} & 0\\ 0 & D_{z,k+1} \end{array}\right],$$

(38) $$D_{x,k+1}=\mathrm{diag}\left(V_{r}\left(e_{k+1}^{\left(1\right)}\right),\ldots ,V_{r}\left(e_{k+1}^{\left(n\right)}\right)\right),$$

(39) $$D_{z,k+1}=\mathrm{diag}\left(V_{r}\left(e_{k+1}^{\left(n+1\right)}\right),\ldots ,V_{r}\left(e_{k+1}^{\left(M\right)}\right)\right),$$

By combining Equation (19), Equation (31), and Equation (36), the following result is obtained:

(40) $${\left(G_{k+1}^{T}D_{k+1}G_{k+1}\right)}^{-1}={\left(S_{k+1}^{-T}D_{x,k+1}S_{k+1}^{-1}+H_{k+1}^{T}S_{r,k+1}^{-T}D_{z,k+1}S_{r,k+1}^{-1}H_{k+1}\right)}^{-1},$$

(41) $$G_{k+1}^{T}D_{k+1}d_{k+1}=S_{k+1}^{-T}C_{x,k+1}S_{k+1}^{-1}{\hat{x}}_{k+1|k}+H_{k+1}^{T}S_{r,k+1}^{-T}D_{z,k+1}S_{r,k+1}^{-1}\left(z_{k+1}-{\hat{z}}_{k+1|k}+H_{k+1}{\hat{x}}_{k+1|k}\right),$$

Furthermore, by combining Equations (36), (40) and (41), the following result is obtained:

(42) $$x_{k+1|k+1}={\hat{x}}_{k+1|k}+K_{k+1}\left(z_{k+1}-{\hat{z}}_{k+1|k}\right),$$

(43) $$K_{k+1}={\bar{P}}_{k+1|k}H_{k+1}^{T}{\left(H_{k+1}{\bar{P}}_{k+1|k}H_{k+1}^{T}+{\bar{R}}_{k+1}\right)}^{-1},$$

where

(44) $${\bar{P}}_{k+1|k}=S_{k+1}D_{x,k+1}^{-1}S_{k+1}^{T},$$

(45) $${\bar{R}}_{k+1}=S_{r,k+1}D_{z,k+1}^{-1}S_{r,k+1}^{T},$$

The corresponding estimation error covariance matrix can be updated using the following equation:

(46) $$P_{k+1|k+1}=P_{k+1|k}-K_{k+1}\left(H_{k+1}P_{k+1|k}H_{k+1}^{T}+R_{k+1}\right)K_{k+1}^{T},$$

## 5. Experiment and Discussion

**Figure 1:**
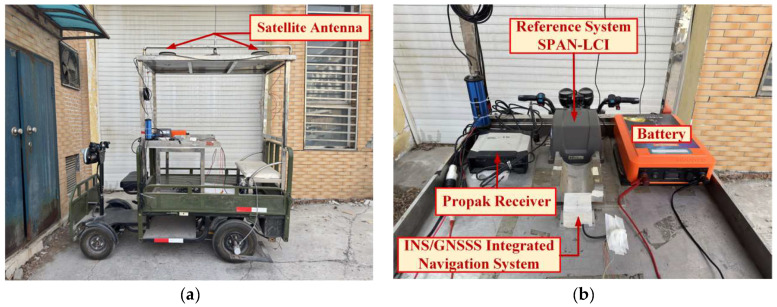
The vehicle experiment platform: (**a**) vehicle platform; (**b**) experimental equipment.

To validate the effectiveness of the proposed adaptive unscented Kalman filtering method based on the MVC for the MEMS-INS/GNSS-integrated navigation system, a vehicular navigation experimental platform was established, and field tests were conducted to evaluate the navigation performance of the proposed method. As shown in Figure 1, the vehicular experimental platform is primarily equipped with the following systems: (1) A MEMS-INS/GPS-integrated navigation system, comprising a triaxial MEMS gyroscope, accelerometer, and GPS receiver, serves as the main experimental system for collecting test data. (2) A NovAtel SPAN-LCI high-precision integrated navigation system, acting as the reference system, provides benchmark information. The specific configurations and key parameters of the relevant systems and sensors are detailed in Table 1.

Based on the aforementioned experimental platform and configuration, the vehicular navigation test was conducted after completing the initial alignment process. The INS/GNSS-integrated navigation system operated in a loosely combination mode, with the GNSS providing velocity and position updates, while the INS delivered high-rate motion measurements. The experiment was performed on the campus of the North University of China, covering a 120 s trajectory, with a total travel distance of approximately 700 m. In this experiment, to evaluate the system’s performance under realistic driving conditions, the test vehicle executed various maneuvers, including sharp turns, straight-line driving, lane changes, and sudden acceleration/deceleration. The road conditions comprised flat, bumpy, uphill, and downhill segments, while the surrounding environment introduced potential GNSS signal degradation due to obstructions such as tall buildings and trees. Under such dynamic driving conditions, the system’s process noise exhibits time-varying statistical characteristics, and the measurement noise occasionally deviates from a Gaussian distribution and may exhibit a non-Gaussian distribution that violate the Gaussian noise assumptions of conventional filtering approaches due to environmental interference. This experiment is mainly aimed at evaluating the performance of the proposed algorithm in complex environments.

The existing extended Kalman filter (EKF), unscented Kalman filter (UKF), and maximum correntropy criterion-based unscented Kalman filter (MCCUKF) are employed as comparison methods to more clearly evaluate the overall performance of the proposed method (MVCAUKF) in the INS/GNSS-integrated navigation system under vehicular test conditions. The Gaussian kernel bandwidth, σ, in the MCCUKF and the versoria radius, r, in the MVCAUKF are critical parameters affecting accuracy and robustness. Taking into account the impact of these parameters on the robustness and computational efficiency, for the MCCUKF, the Gaussian kernel bandwidth was set to σ=10, and for the proposed method, the versoria radius was set to r = 10. All algorithms were executed on Intel Core i7-11800H processors with 2.3 GHz and a 16 GB RAM configuration.

**Figure 2:**
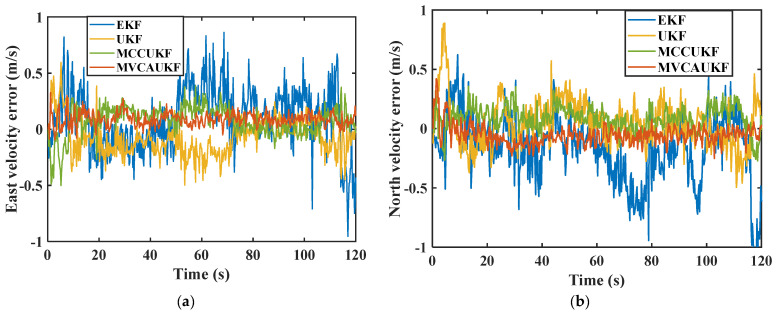
Velocity errors of the various algorithms: (**a**) east velocity error; (**b**) north velocity error.

**Figure 3:**
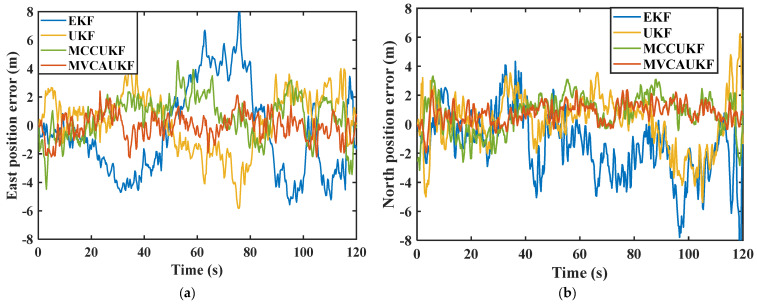
Position errors of the various algorithms: (**a**) east position error; (**b**) north position error.

Figure 2 compares the east and north velocity errors obtained by the four different algorithms, and the east and north position errors obtained by the four different algorithms are shown in Figure 3. Furthermore, Table 2 summarizes the root mean square error (RMSE) of the east and north positions and velocities for the different filtering algorithms, providing a quantitative comparison of the navigation algorithms. The RMSE quantifies the deviation between the estimated values and the reference data, exhibiting heightened sensitivity to outliers within the dataset. A smaller RMSE value indicates superior navigational accuracy, reflecting greater robustness against anomalous measurements. In our paper, the RMSE can be defined as follows:

(47) $$\mathrm{RMSE}=\sqrt{\frac{1}{N}\sum_{k=1}^{N}{\left(P_{k}-{\hat{P}}_{k}\right)}^{2}},$$

where N denotes the total sample number, $p_{k}$ and ${\hat{p}}_{k}$ represent the reference value provided by the reference system and the estimated value solved by the navigational algorithm of velocity, position, and attitude at time k.

From the above comparison results, it can be seen that in complex navigation environments, the proposed adaptive Kalman filter algorithm based on maximum versoria criterion demonstrates better navigation performance compared to other methods. In the face of complex navigation environments and noise interference, the traditional EKF algorithm does not incorporate corresponding measures to address system process uncertainty and complex measurement noise. Simple linearization cannot effectively solve strong nonlinear problems, resulting in the poorest navigation performance among the four methods. The standard UKF algorithm is relatively sensitive to system process uncertainty and non-Gaussian measurement noise. Due to the lack of effective suppression measures for noise characteristic changes caused by complex environments, the navigational performance is not ideal and the error is relatively large. Both the maximum correntropy criterion-based unscented Kalman filter (MCCUKF) and the proposed adaptive unscented Kalman filter based on the maximum versoria criterion (MVCAUKF) can effectively cope with complex processes and noise. Furthermore, compared to the existing MCCUKF, the proposed method fully considers the anomalous measurement information induced by complex environments and adopts the versoria function, which has a simpler form and is more suitable for handling non-Gaussian measurement noise. This enables the proposed method to effectively mitigate the impact of non-Gaussian measurement noise on the integrated filter, demonstrating better robustness and anti-interference capabilities. Thereby achieving better navigation parameter estimation results and obtaining more accurate navigation information. Compared to the EKF, UKF, and MCCUKF, the proposed method reduced the RMSE of the east velocity by 70.96%, 57.14%, and 43.75%, respectively, and the RMSE of the north velocity by 65.52%, 54.54%, and 33.33%, respectively. The east position error was reduced from 3.23 m, 2.33 m, and 1.78 m to 0.91 m, improving the accuracy by 71.82%, 60.94%, and 48.88%, respectively. Similarly, the north position error was reduced from 2.96 m, 2.12 m, and 1.63 m to 1.01 m, improving the accuracy by 65.87%, 52.36%, and 38.04%, respectively. These results demonstrate that the proposed method exhibits a superior navigational performance. It is also evident that, in complex navigation environments, the proposed method can maintain a good estimation performance and remain robust for an integrated navigation system with uncertain process noise.

In terms of computational costs, Table 3 compares the running time of each method. It is noted that the computational time provided in Table 3 represents the total running time for processing the entire dataset in the vehicular navigation experiment (120 s of trajectory data), not the time per iteration. From Table 3, it can be clearly seen that the MCCUKF and MVCAUKF exhibited significantly improved navigational accuracy and robustness at the cost of increased computations. However, compared with the MCCUKF, the proposed MVCAUKF method not only achieves higher accuracy and greater robustness but also a shorter computation time due to the use of a more concise maximum versoria function.

**Figure 4:**
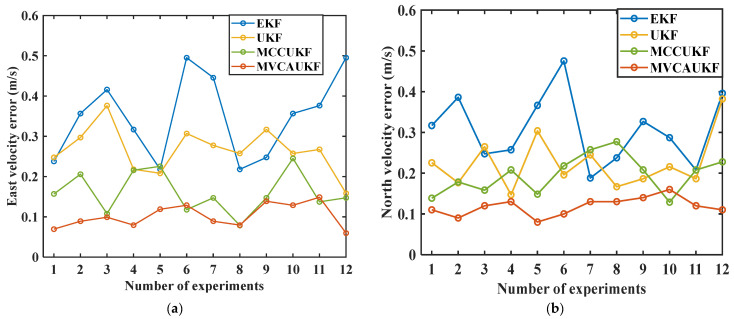
Velocity errors from the various methods in experiments 1–12: (**a**) east velocity error; (**b**) north velocity error.

**Figure 5:**
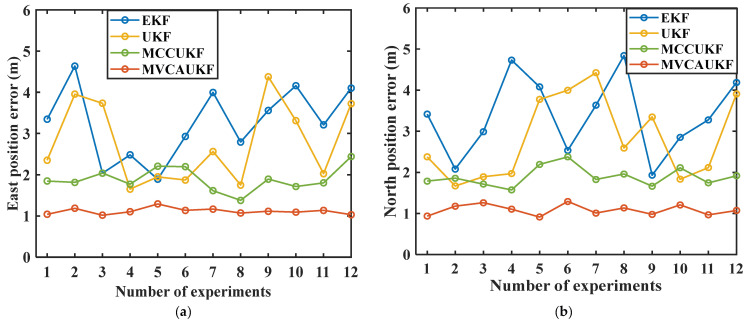
Position errors from the various methods in experiments 1–12: (**a**) east position error; (**b**) north position error.

To eliminate the uncertainty arising from a single experiment run, we performed 12 repeated experiments following the established experiment scheme, with the same configuration and similar vehicle driving trajectory for each experiment. The velocity and position errors, quantified by the RMSE, were calculated for each method across all 12 experiments. The RMSE velocity errors and position errors obtained from the various methods are presented for experiments 1–12, which were conducted according to the experiment scheme, in Figure 4 and Figure 5.

Based on the experimental data presented in Figure 4 and Figure 5, it can be seen that the proposed MVCAUKF method demonstrated relatively high navigational accuracy and output relatively stable results in multiple similar experiments. These findings also indicate the method’s good performance in handling complex navigation conditions.

## 6. Conclusions

To enhance the navigational performance of INS/GNSS-integrated systems in complex environments, this study focused on addressing the issues of system uncertainties caused by measurement anomalies and the impact of non-Gaussian measurement noise. An INS/GNSS-integrated navigation method based on the maximum-versoria-criterion adaptive unscented Kalman filter is proposed. By constructing an adaptive unscented Kalman filter framework and formulating a cost optimization function based on the maximum versoria criterion, the proposed method effectively mitigates the negative effects of measurement anomalies and non-Gaussian measurement noise on system navigation performance. The vehicular navigation experiments demonstrate that the proposed method can maintain good navigational accuracy and robustness in complex environments. Future studies will focus on advanced noise characterization methods to explicitly model the non-Gaussian and time-varying properties of the GNSS measurement noise in complex environments, thereby refining the adaptive mechanisms of the proposed algorithm.

## Figures and Tables

**Table 1:** Parameters of the relevant navigation systems.

System	Parameter	Value
INS	Gyroscope bias	1°/h
	Gyroscope random walk	0.5°/ $\sqrt{h}$
	Accelerometer bias	5 mg
GNSS	Positioning accuracy	2 m
	Speed accuracy	0.2 m/s
	Time accuracy	50 ns
Reference system (NovAtel SPAN-LCI)	Positioning accuracy	1 cm ± 1 ppm
	Speed accuracy	0.03 m/s
	Time accuracy	20 ns

**Table 2:** RMSE of velocity errors and position errors for various methods.

Method	East Velocity Error/(m/s)	North Velocity Error/(m/s)	East Position Error/(m)	North Position Error/(m)
EKF	0.31	0.29	3.23	2.96
UKF	0.21	0.22	2.33	2.12
MCCUKF	0.16	0.15	1.78	1.63
MVCAUKF	0.09	0.10	0.91	1.01

**Table 3:** Computational costs of the various methods.

Method	EKF	UKF	MCCUKF	MVCAUKF
Computation time (s)	12.56	19.03	28.23	23.27

## Data Availability

The original contributions presented in this study are included in the article. Further inquiries can be directed to the corresponding author.
